# Supplementary material for: Differences between Arterial and Venous Umbilical Cord Plasma Metabolome and Association with Parity
Source: Metabolites. 2022 Feb 13;12(2):175. doi: 10.3390/metabo12020175 (PMC8877791; doi:10.3390/metabo12020175)
Supplement: Supplementary file 1 [file metabolites-12-00175-s001.zip › metabolites-1543037-supplementary.pdf]

Supplementary information

Table S1. List of all annotated metabolites used for the study, along with their HMDB ID where applicable.

Metabolite	HMDB ID
Diaminopropane	HMDB0000002
1,5-Anhydro-d-mannitol	HMDB0244228
Cadaverine	HMDB0002322
1-Aminocyclopropanecarboxylic acid	HMDB0036458
3-Palmitoyl-sn-glycerol	HMDB0245964
1-Palmitoyl-sn-glycero-3-phosphocholine derivative	NA
Homogentisic acid	HMDB0000130
2-Amino-2-methyl-1-propanol	HMDB0244974
D-alpha-Aminobutyric acid	HMDB0000650
Citramalic acid	HMDB0000426
3,4-Dihydroxybenzeneacetic acid	HMDB0001336
3-Hydroxyphenylacetic acid	HMDB0000440
Hydroxypyruvic acid	HMDB0001352
3-Oxoglutaric acid	HMDB0013701
gamma-Aminobutyric acid	HMDB0000112
4-Hydroxyphenyl acetate	HMDB0060390
5-Aminolevulinic acid	HMDB0001149
Abscisic acid	HMDB0035140
Acetaminophen	HMDB0001859
Adenosine monophosphate	HMDB0000045
Adipic acid	HMDB0000448
L-Allothreonine	HMDB0004041
Benzoic acid	HMDB0001870
d-beta-homoglutamine	NA
Creatinine	HMDB0000562
DL-Cysteine	HMDB0251515
Deoxycholic acid	HMDB0000626
Docosahexaenoic acid	HMDB0002183
Docosapentaenoic acid (22n-6)	HMDB0001976
Dodecanoic acid	HMDB0000638
Eicosapentaenoic acid	HMDB0001999
Eicosatetraenoic acid	HMDB0251721
Erythritol	HMDB0002994
Ethanolamine	HMDB0000149
DL-Ethionine	HMDB0251518
Fructose 1,6-bisphosphate	HMDB0001058
Galacturonic acid	HMDB0002545
gamma-Glutamylphenylalanine	HMDB0000594
Gibberellin A1	NA
Glucaric acid	HMDB0000663
d-Glucoheptose	NA

(Z)-4-Hydroxy-6-dodecenoic acid lactone	HMDB0032331
Gluconolactone	HMDB0000150
D-Glucuronic acid	HMDB0000127
L-Glutamine	HMDB0000641
Glutaric acid	HMDB0000661
2-Phosphoglyceric acid	HMDB0000362
Glyceric acid	HMDB0000139
Glycolic acid	HMDB0000115
Glycylvaline	HMDB0028854
Heptadecanoic acid	HMDB0002259
Pimelic acid	HMDB0000857
Palmitic acid	HMDB0000220
Hippuric acid	HMDB0000714
Homocysteine	HMDB0000742
Hypoxanthine	HMDB0000157
Indoleacetic acid	HMDB0000197
scyllo-Inositol	HMDB0006088
Isocitric acid	HMDB0000193
Isoerythritol	NA
Maleic acid	HMDB0000176
Malic acid	HMDB0000744
Mannitol	HMDB0000765
Acetylcysteine	HMDB0001890
N-Acetyl-D-glucosamine	HMDB0000215
N-Acetylmannosamine	HMDB0001129
N2-Acetylornithine	HMDB0003357
Nicotinic acid	HMDB0001488
Pelargonic acid	HMDB0000847
L-Norleucine	HMDB0001645
Linoleic acid	HMDB0000673
Stearic acid	HMDB0000827
gamma-Linolenic acid	HMDB0003073
alpha-Linolenic acid	HMDB0001388
Elaidic acid	HMDB0000573
Oleic acid	HMDB0000207
O-Phosphoethanolamine	HMDB0000224
Oxalic acid	HMDB0002329
Oxalacetic acid	HMDB0000223
Pentadecanoic acid	HMDB0000826
Phenylpyruvic acid	HMDB0000205
Pyridoxal	HMDB0001545
Pyroglutamic acid	HMDB0000267
Pyruvic acid	HMDB0000243
Quinic acid	HMDB0003072
ribose-5-phosphate	HMDB0001548
Sarcosine	HMDB0000271
Sinapaldehyde	HMDB0258301
Sorbitol	HMDB0000247

L-Tryptophan	HMDB0000929
Undecanoic acid	HMDB0000947
Urea	HMDB0000294
Uric acid	HMDB0000289
D-Alanine	HMDB0001310
2-Hydroxybutyric acid	HMDB0000008
3-Hydroxybutyric acid	HMDB0000357
3-Methyl-2-oxovaleric acid	HMDB0000491
Ketoleucine	HMDB0000695
D-Valine	HMDB0250806
D-Leucine	HMDB0013773
L-Proline	HMDB0000162
L-Isoleucine	HMDB0000172
Glycine	HMDB0000123
Succinic acid	HMDB0000254
Itaconic acid	HMDB0002092
Fumaric acid	HMDB0000134
L-Serine	HMDB0000187
L-Threonine	HMDB0000167
beta-Alanine	HMDB0000056
L-Methionine	HMDB0000696
4-Hydroxyproline	HMDB0000725
Oxoglutaric acid	HMDB0000208
L-Glutamic acid	HMDB0000148
L-Phenylalanine	HMDB0000159
DL-Asparagine	HMDB0251512
cis-Aconitic acid	HMDB0000072
Ornithine	HMDB0000214
Dimethylarginine	HMDB0251395
L-Histidine	HMDB0000177
L-Lysine	HMDB0000182
Inosine	HMDB0000195
gamma-Tocopherol	HMDB0001492
alpha-Tocopherol	HMDB0001893
Cholesterol	HMDB0000067

Table S2. List of internal standards

**Internal Standard name**
L-Glutamic acid 13C5; 15N
alpha-Ketoglutaric acid 13C4
2-Hydroxybenzoic acid D6
Myristic acid 13C3
Cholesterol D7

Palmitic acid 13C4

L-Proline 13C5

1,4-Butanediamine D4

Succinic acid D4

D-Glucose 13C6

Table S3. Settings for targeted data acquisition.

Target Settings	
RI low	40
RI high	40
Peak Width(s)	1
Smooth Filter Length	15
Target Num Scans	3
Spectrum Correlation	0.8
Target Correlation	0.8
Single M/z Correlation	0.7
Model Settings	---
Peak Treshold Level	0.02
Peak Split Level	0.5
Savgol Filter Length	31
Peak Width Min	30
Peak Width Max	150
Max Num Peaks	4
Unimodal Threshold	0.975
Processing Settings	---
Processing Method	Target
Training Set Method	Automatic
Training Set Samples	20
Prediction Set Samples	269
Recursion	On
Unimodal Modelling	On
